# Pesticide Contamination in the Hair of Children From Colonia San Juan, a Rural Community in Paraguay

**DOI:** 10.1002/dta.70020

**Published:** 2025-12-26

**Authors:** Stela Benitez Leite, Alba Iglesias‐Gonzalez, Mirta Noemí Mesquita, María Luisa Macchi, Robin Mesnage, Brice M. R. Appenzeller

**Affiliations:** ^1^ Facultad de Ciencias de la Salud Universidad Católica Nuestra Señora de la Asunción Asunción Paraguay; ^2^ Human Biomonitoring Research Unit, Department of Precision Health Luxembourg Institute of Health Strassen Luxembourg; ^3^ Hospital General Pediatrico Niños de Acosta Ñu San Lorenzo Paraguay; ^4^ Department of Nutritional Sciences, School of Life Course Sciences, Faculty of Life Sciences and Medicine King's College London London UK

**Keywords:** biomonitoring in hair, children, pesticides

## Abstract

Chronic exposure to pesticides can cause carcinogenic, reproductive, neurological, and endocrine‐disrupting effects. Hair analysis is a valuable biomonitoring tool to assess human exposure to pesticides. We determined the presence of pesticides, their metabolites, and other environmental pollutants in the hair of children in an agricultural area of Paraguay. We analyzed 152 pesticides and environmental chemicals in hair samples from 51 children (2–14 years, mean ± SD = 8.5 ± 3.3 years) living in Colonia San Juan, a rural community in Paraguay. The locality is surrounded by soybean crops, and the community engages primarily in family farming. Eighty of the 152 compounds (52.6%) were detected. Each child's sample contained an average of 55 ± 3.7 compounds (range 48–65), including organophosphates, pyrethroids, neonicotinoids, fungicides, herbicides, and endocrine disruptors such as bisphenol A and bisphenol S. Thirty‐seven compounds were present in all samples. Children in this rural community are simultaneously exposed to numerous pesticides and pollutants, highlighting the urgent need for stricter environmental protections and preventive health measures.

## Graphical abstract

Hair analysis in children from a rural community in Paraguay reveals exposure to organophosphates, pyrethroids, neonicotinoids, fungicides, herbicides, and endocrine disruptors, highlighting the urgent need for stricter environmental protections and preventive health measures.

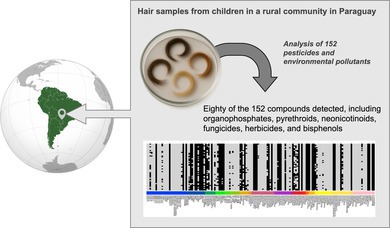

## 1 Introduction

Human activities have led to widespread contamination of air, water, and soil, contributing to the development of chronic diseases [1]. Pesticide exposure occurs both directly, during agricultural and livestock management [2], and indirectly, exposed through the ingestion of contaminated food [3, 4], water [5], or by inhalation of pesticide droplets dispersed in the environment [6].

Pesticides are integral to modern agriculture for pest control and yield optimization, yet they are major contributors to the chronic disease burden because many act on the nervous [7] and endocrine systems [8]. In pediatric populations, exposure to pesticides has been associated with neurodevelopmental disorders and potentially an increased risk of cancer [9, 10].

The ingredients in pesticide formulations often act in mixtures, and their cumulative effects, commonly referred to as the “cocktail effect,” are not always accurately predicted by current regulatory frameworks, which typically focus on individual compounds in isolation [11]. This gap underscores the importance of biomonitoring to evaluate real‐world exposure, especially among populations residing near intensive agricultural areas.

Biomonitoring can rely on biological matrices such as urine or hair. Among these, hair analysis is particularly valuable: It provides a stable, noninvasive, and easily stored record of both acute and chronic exposure to drugs, metals, and organic pollutants [12, 13].

In Paraguay, the introduction of genetically modified soybeans in 2004 significantly increased pesticide use, especially in the major soybean‐producing departments of Alto Paraná, Itapúa, and Canindeyú [14, 15]. This increase in pesticide use accompanied the expansion of soybean cultivation areas [16]. Currently, Paraguay ranks sixth in soybean production and third in soybean exports globally [17]. The growing use of pesticides and the lack of enforcement of protective regulations have been corroborated by a UN rapporteur sent to Paraguay in response to complaints about adverse health effects caused by pesticides [18].

**FIGURE 1:**
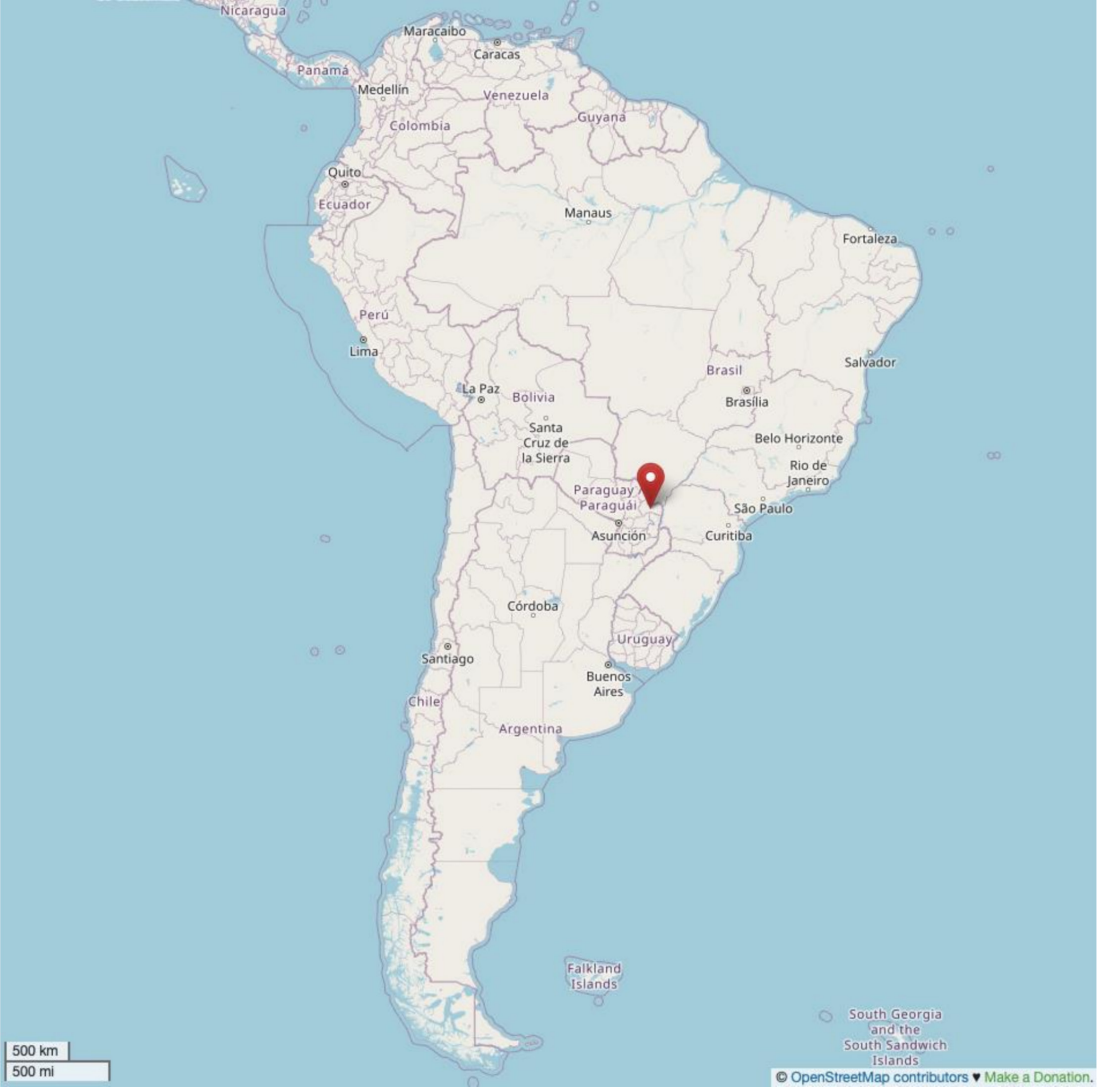
Localization of Colonia San Juan, a rural locality in Francisco Caballero Álvarez district, in the Department of Canindeyú in Paraguay. Map from OpenStreetMap (https://www.openstreetmap.org/).

In a prior study conducted in Colonia San Juan (24°08′03.0″S 54°46′59.3″W) (Figure 1), a rural area in the Francisco Caballero Álvarez district of Canindeyú surrounded by transgenic soybean crops that fail to comply with protection laws whose inhabitants are engaged in family farming [18], one of the authors identified genotoxic and cytotoxic effects in children aged 5 to 10 years [19].

Building on these findings, a second study was conducted 2 years later in the same community. Using hair biomonitoring, it aimed to identify pesticides, their metabolites, and other environmental contaminants in children aged 2–14 years, providing a detailed snapshot of chronic chemical exposure in this vulnerable population.

## 2 Material and Methods

### 2.1 Ethics

The work was carried out respecting the ethical principles of research. The Ethics Committee of the Catholic University approved the protocol. We carefully respected the principles of Respect for Persons, Beneficence, and Justice. At all times, we guaranteed free participation (Declaration of Helsinki) and data protection through the confidentiality of the interviewees (use of alphanumeric codes). Children whose parents gave informed consent to participate were included in the study by non‐probabilistic sampling at the convenience of the researchers.

### 2.2 Study Design, Population and Site of Recruitment

The study was prospective, observational, descriptive, and cross‐sectional. The participants were inhabitants of Colonia San Juan, a rural locality in Francisco Caballero Álvarez district, in the Department of Canindeyú in Paraguay. This population is dedicated to family farming, and the area of residence is surrounded by soybean crops. In November 2018, the community leaders made a call to parents of children attending the San Roque School No. 4301 in Colonia San Juan to introduce them to the research team and explain the objective of the study. Before entering the study, parental consent was requested. The included population underwent a clinical examination, carried out by one of the researchers. Carriers of congenital malformations were excluded. Demographic variables (age and sex) were collected through a data sheet.

### 2.3 Sample Collection

After the physical examination, one hair sample was taken from each child by using scissors, cutting a strand of hair from the occipital region, as close as possible to the scalp. The hair were 5.1 ± 2.8 cm long. This hair sample was immediately wrapped in aluminum foil and transported to the laboratory for the corresponding examination.

Each hair sample was washed with three successive washings of (a) sodium dodecyl sulfate (SDS) (Reagent Plus*L4509, Sigma‐Aldrich), (b) ultrapure water (Millipore‐AFS‐8 system), and (c) methanol (Biosolve‐Analytical grade), under agitation, following the validated protocol described in Duca et al. [20]. The samples were then placed on a tissue, gently rubbed, and placed under a gentle airflow to dry. Once the samples were dried, they were placed in a stainless steel jar for pulverization using a Retsch MM200 ball mill at 25 Hz and 50 mg of hair powder per sample was placed in 4‐mL screw‐neck glass vials (La‐Pha‐Pack).

### 2.4 Chemical Extraction and Analysis

The methodology used in the chemical analysis explained below has been published and fully validated by Beranger et al. [21]. Briefly, each sample was supplemented with 10 μL of internal standard solution (stable isotope‐labeled analogs from Dr. Ehrenstorfer, Sigma‐Aldrich, Toronto Research Chemicals [Toronto, ON, Canada], Cambridge Isotope Laboratories [Tewkesbury, MA, USA], and US Biological [Swampscott, MA, USA]) and 1 mL of an acetonitrile–water mixture (50/50) (Biosolve‐ULC/MS grade), and placed in a New Brunswick‐G25 incubator shaker at 37°C with stirring at 350 rpm for 12 h. After 12 h of extraction, samples were centrifuged for 10 min at 2800 *g* (in a Sigma 4–16 KS centrifuge) and the supernatant was then divided to test for the presence of nonpersistent and persistent organic pollutants (POPs).

To test for the presence of non‐POPs, 200 μL of the recovered extract was evaporated under a gentle nitrogen stream and reconstituted into 50 μL of a 10‐mM ammonium acetate acetonitrile buffer solution. The samples were then centrifuged for 5 min at 18,000 *g* (in a Sigma 1–16K centrifuge), and after that, the supernatants were recovered and placed in 2‐mL screw‐neck vials (La‐Pha‐Pack) for injection into a liquid chromatography coupled to tandem mass spectrometer (LC–MS/MS) (Waters Atlantis).

To analyze POPs, 300 μL of extract were placed in 10‐mL screw neck glass vial with metal caps (Supelco) with 7.6 mL of phosphate buffer 1 M. Samples were placed on a GC–MS/MS (Agilent Technologies 7000A model) to perform a solid phase micro extraction (SPME). The chromatographic and mass spectrometric conditions were fully detailed in previous articles [13, 21]. All validation details are made available (Table S1).

### 2.5 Data Processing and Analysis

The data were exported from the Excel spreadsheet to the SPSS V 21 (IBM USA) statistical package. The qualitative variables were expressed as percentages, whereas the quantitative variables were expressed as median with quartiles or means with standard deviation according to their distribution determined by the Kolmogorov–Smirnov test.

We estimated the role of age and sex. When the proportion of detected metabolites was over 50%, which was the case for 51 metabolites, statistical significances were evaluated with a linear regression model with R version 4.0.0.

## 3 Results

**FIGURE 2:**
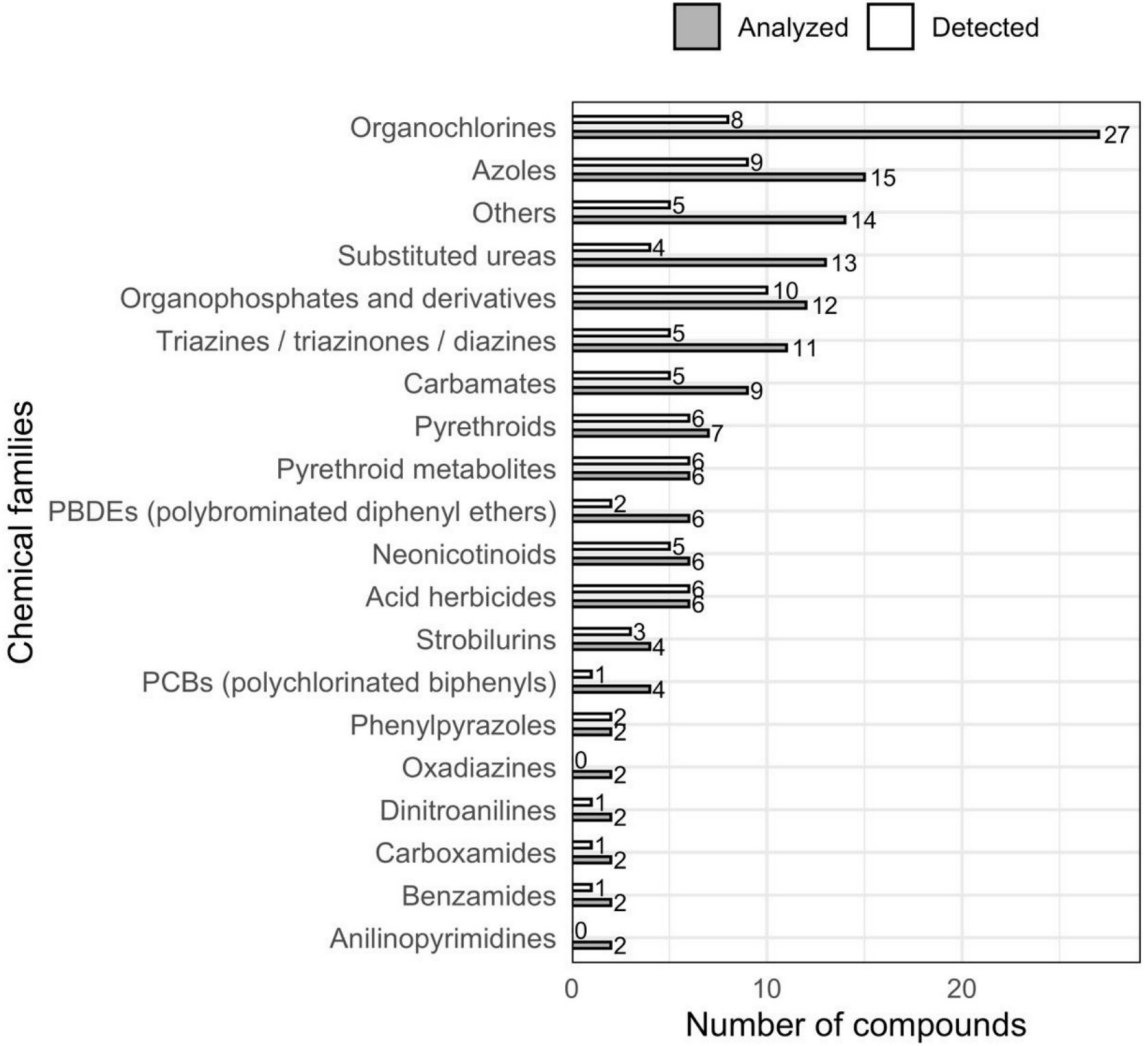
Number of chemical compounds studied (*n* = 152) (gray bars) and detected (*n* = 80) (white bars) in the hair samples analyzed, categorized by chemical families.

**FIGURE 3:**
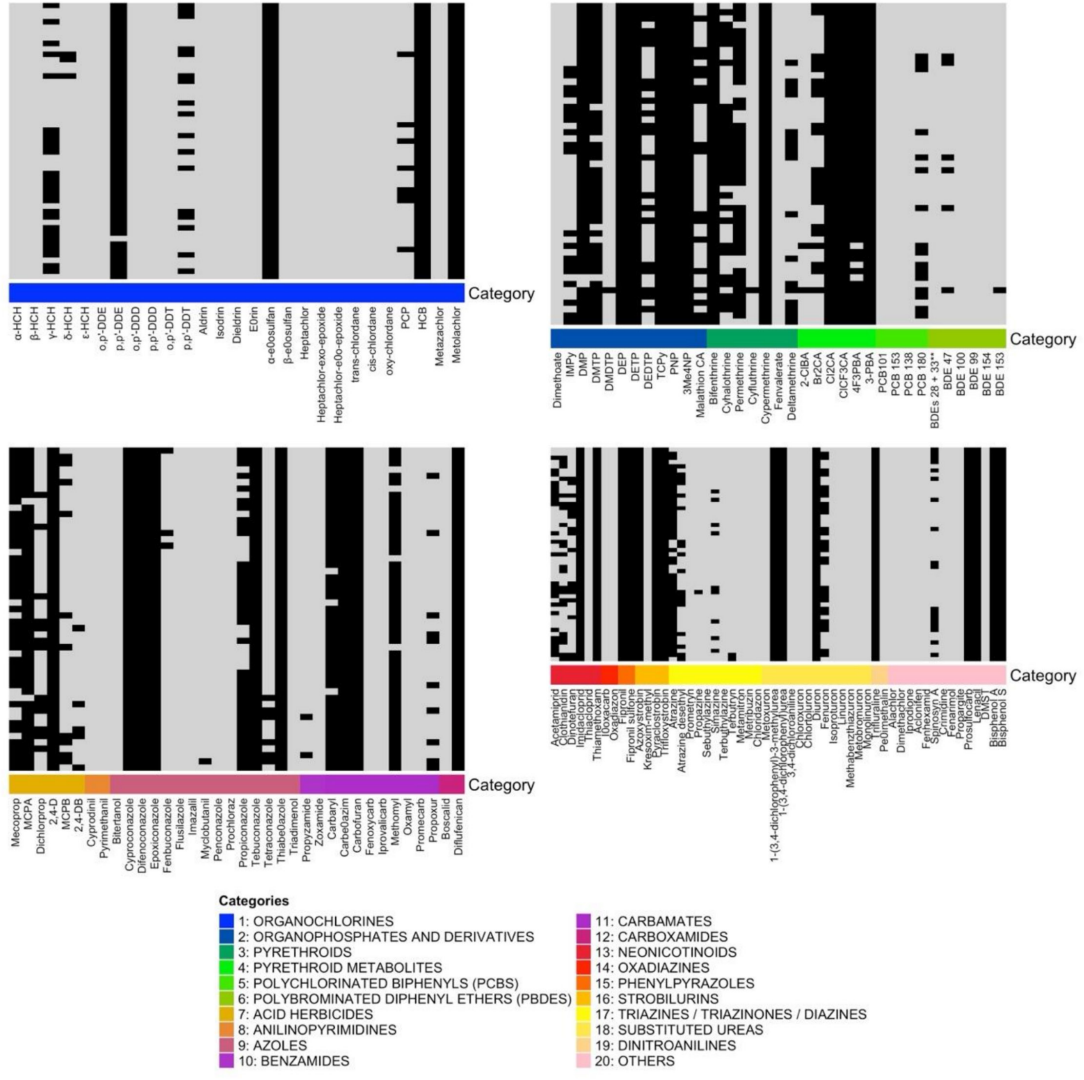
Presence of environmental pollutants in their hair samples of 51 children from the San Juan Colony. Presence (black) or absence (gray) of the different compounds is displayed as a heatmap for the different children. Categories for the different chemicals measured are also displayed as a color code.

A total of 51 children with a mean age of 8.5 ± 3.3 years, 56.9% (29/51) males, entered the study. One hair sample was collected per participant, with a mean length of 5.1 ± 2.8 cm. Each sample was analyzed for 152 chemical compounds spanning 19 classes of environmental pollutants, including pesticides (Figure 2). Overall, 81 of the 152 compounds (53.2%) were detected (Table 1). On average, each hair sample contained 55 ± 3.7 compounds (range: 48–65). Of all biomarkers tested, 37 compounds were present in 100% of samples (Figure 3). Among the chemical compounds detected in 100% of the samples, the following pesticide families were found:

- Pyrethroids and metabolites (bifenthrin, cypermethrin, Cl_2_CA, ClCF_3_CA, 3‐PBA)
- Neonicotinoids (thiamethoxam, imidacloprid)
- Phenylpyrazoles (fipronil, fipronil sulfone)
- Carbamates (carbendazim)
- Organophosphates (3Me4NP, PNP, TCPy [chlorpyrifos], DEP, DETP)
- Organochlorines (α‐endosulfan, hexachlorobenzene [HCB], metolachlor)
- Herbicides (2,4‐D, lenacil, prosulfocarb)
- Urea‐derived fertilizers (diuron and related compounds)
- Fungicides of the azole class (cyproconazole, epoxiconazole, difenoconazole)
- Strobilurins (trifloxystrobin, azoxystrobin, pyraclostrobin)
- Endocrine disruptors (bisphenol A, bisphenol S).

**TABLE 1:** Demographic variables of the pediatric population studied. Values are indicated as mean ± SD.

Variable	Value
Male (%)	29 (56.9)
Females (%)	22 (43.1)
Age (years)	8.5 ± 3.3
Hair Length (cm)	5.1 ± 2.8

The highest median concentrations were observed for bifenthrin (median: 61 pg/mg hair), permethrin (median: 34‐pg hair), cypermethrin (29 pg/mg hair), 2,4‐D (20 pg/mg hair), and bisphenol S (median: 9‐pg hair) (Table 2). Other compounds occurred at lower levels. Some, such as α‐endosulfan and HCB, were detected below their limits of quantification in all samples (Table 2).

**TABLE 2:** Description of quartile concentrations in the hair samples studied (*n* = 51), according to the chemical compounds detected.

	*n*	%	Perc_25 (pg/mg)	Perc_50 (pg/mg)	Perc_75 (pg/mg)	Min (pg/mg)	Max (pg/mg)	LOQ (pg/mg)
Organochlorines								
α‐HCH	—	—	—	—	—	—	—	0.5
β‐HCH	—	—	—	—	—	—	—	1
ε‐HCH	—	—	—	—	—	—	—	0.5
γ‐HCH	26	51	0.09	0.11	0.15	0.06	0.76	0.32
δ‐HCH	3	5.9	0.48	1.57	—	0.48	2.5	1
p,p′‐DDE	50	98	4.5	6.8	9.8	1.3	361	0.5
p,p′‐DDT	17	33	3.6	5.9	14.9	2.8	22.7	2
o,p′‐DDE	—	—	—	—	—	—	—	0.5
o,p′‐DDD	—	—	—	—	—	—	—	2
p,p′‐DDD	—	—	—	—	—	—	—	5
o,p′‐DDT	—	—	—	—	—	—	—	5
Aldrin	—	—	—	—	—	—	—	0.5
Isodrin	—	—	—	—	—	—	—	0.5
Dieldrin	—	—	—	—	—	—	—	0.14
Endrin	—	—	—	—	—	—	—	0.5
α‐Endosulfan	51	100	0.12	0.16	0.19	0.04	2.1	1
β‐Endosulfan	—	—	—	—	—	—	—	0.5
Heptachlor	—	—	—	—	—	—	—	0.5
Heptachlor‐exo‐epoxide	—	—	—	—	—	—	—	1
Heptachlor‐e o‐epoxide	—	—	—	—	—	—	—	0.5
*Trans*‐chlordane	—	—	—	—	—	—	—	0.5
*Cis*‐chlordane	—	—	—	—	—	—	—	0.5
*Oxy*‐chlordane	—	—	—	—	—	—	—	0.5
PCP	8	15.7	0.23	0.68	1.22	0.14	1.61	0.5
HCB	51	100	0.04	0.05	0.06	0.03	0.09	0.19
Metazachlor	—	—	—	—	—	—	—	0.5
Metolachlor	51	100	0.01	0.16	0.29	0.07	0.5	0.5
Organophosphate								
3Me4NP	51	100	2.4	3.6	9.5	0.66	170	0.5
DEDTP	21	41	0.02	0.04	0.62	0.01	5	10
PNP	51	100	8.8	13.6	21.1	3.7	42	5
TCPy	51	100	0.83	1.8	3.2	0.31	38.3	0.5
Malathion CA	18	35	1.2	2.1	3.4	0.72	4.8	0.5
DEP	51	100	0.74	1.3	2.5	0.09	15	2
DETP	51	100	0.13	0.22	0.44	0.07	1.8	0.5
Dimethoate	—	—	—	—	—	—	—	0.5
DMP	—	—	—	—	—	—	—	—
DMTP	45	88	0.13	0.19	0.69	0.04	51.2	1
DMDTP	1	2	0.16	0.16	0.16	0.16	0.16	—
IMPy	19	37	0.19	0.29	0.92	0.1	11.7	0.5
Pyrethroids								
Bifenthrin	51	100	16	61.6	203	2.2	952	1
Cypermethrin	51	100	7.9	26.7	84.6	1.8	24,769	4
Permethrin	40	78	8.7	34.6	117	4	130,459	2.5
Cyhalothrin	29	57	2.4	16.3	39.7	0.7	1023	0.5
Deltamethrin	17	33	2.8	6.2	17.3	0.3	111	0.5
Fenvalerate	—	—	—	—	—	—	—	1.92
Cyfluthrin	1	2	5	5	5	5	5	2
Pyrethroid metabolites								
2‐ClBA	2	3.9	0.13	0.36	—	0.13	0.59	0.5
Br2CA	22	43	0.06	0.12	0.48	0.04	25.2	0.5
Cl2CA	51	100	2.8	6.3	19.1	1.5	2211	1
4F3PBA	48	94	0.04	0.06	0.1	0.02	1.75	0.5
CICF3CA	51	100	0.76	1.8	3.7	0.04	33.9	—
3‐PBA	51	100	6.2	11.6	18.6	1.5	1553	1
PCBs								
PCB101	—	—	—	—	—	—	—	5
PCB 153	—	—	—	—	—	—	—	2
PCB 138	—	—	—	—	—	—	—	0.5
PCB 180	16	31	1.6	2.5	3.7	0.74	14.1	0.5
PBDEs								
BDEs 28 + 33	—	—	—	—	—	—	—	0.5
BDE 47	6	12	1.2	2.6	8.5	0.76	10.6	2
BDE 100	—	—	—	—	—	—	—	10
BDE 99	—	—	—	—	—	—	—	5
BDE 154	—	—	—	—	—	—	—	1
BDE 153	1	2	3.7	3.7	3.7	3.7	3.7	2
Acidic herbicides								
Mecoprop	41	80	0.13	0.21	0.37	0.08	8.7	2
MCPA	49	96	0.16	0.19	0.22	0.12	1.4	2
Dichlorprop	13	26	0.15	0.37	2	0.03	23.7	0.5
2,4‐D	51	100	6.1	21	70.2	0.77	1747	0.5
MCPB	10	20	0.1	0.23	0.33	0.04	1.7	0.5
2,4‐DB	5	9.8	0.04	0.04	0.93	0.04	1.8	0.5
Azoles								
Bitertanol	—	—	—	—	—	—	—	2
Cyproconazole	51	100	4.8	10	18.7	0.75	86.5	0.5
Difenoconazole	51	100	1.8	4	6.8	0.27	22.9	0.5
Epoxiconazole	51	100	1.6	4.8	10.2	0.47	23.2	0.5
Fenbuconazole	3	5.9	0.08	0.18	—	0.08	0.29	1
Flusilazole	—	—	—	—	—	—	—	0.5
Imazalil	—	—	—	—	—	—	—	2
Myclobutanil	1	2	0.32	0.32	0.32	0.32	0.32	0.5
Penconazole	—	—	—	—	—	—	—	0.5
Prochloraz	—	—	—	—	—	—	—	0.5
Tebuconazole	51	100	2.3	4.9	9	0.32	52.2	0.5
Propiconazole	37	73	0.16	0.26	0.45	0.03	2	0.3
Thiabendazole	51	100	0.27	0.72	2.3	0.04	77	0.25
Tetraconazole	6	12	0.92	1	2.2	0.86	3.8	0.5
Triadimenol	—	—	—	—	—	—	—	5
Benzamides								
Zoxamide	—	—	—	—	—	—	—	0.5
Propyzamide	2	3.9	0.29	0.53	—	0.29	0.77	2
Carbamates								
Carbendazim	51	100	10	18	38	3.3	358	1
Carbofuran	51	100	0.23	0.43	0.66	0.04	12.6	0.5
Fenoxycarb	—	—	—	—	—	—	—	0.5
Iprovalicarb	—	—	—	—	—	—	—	0.5
Methomyl	47	92	0.41	0.59	1.1	0.07	15.3	0.5
Carbaryl	48	94	1.2	2.3	16	0.4	557	0.1
Oxamyl	—	—	—	—	—	—	—	0.5
Promecarb	—	—	—	—	—	—	—	0.5
Propoxur	10	20	0.41	0.59	1.1	0.07	15.3	0.5
Carboxamides								
Boscalid	—	—	—	—	—	—	—	0.5
Diflufenican	51	100	0.15	0.2	0.32	0.09	27.5	0.5
Neonicotinoids								
Acetamiprid	24	47	0.08	0.17	0.42	0.04	3	0.5
Clothianidin	22	43	0.2	0.28	0.73	0.11	2.9	0.5
Dinotefuran	17	33	1.2	1.9	4	0.35	7.5	0.5
Imidacloprid	51	100	1.5	2.8	10	0.68	142	0.5
Thiacloprid	—	—	—	—	—	—	—	0.5
Thiamethoxam	51	100	1.7	4.2	10.1	0.58	223	0.5
Phenylpyrazoles								
Fipronil	51	100	1.9	6.7	45	0.52	379	0.5
Fipronil sulfone	51	100	3.6	7.3	26	0.8	139	1
Strobilurins								
Azoxystrobin	51	100	3.7	8.4	24	0.68	200	0.5
Kresoxim‐methyl	—	—	—	—	—	—	—	0.5
Pyraclostrobin	51	100	0.84	2.3	4.5	0.36	30.7	1
Trifloxystrobin	51	100	3.6	7.8	14.8	1.4	46.1	0.5
Triazines/triazinones/diazines								
Atrazine	43	84	0.19	0.53	0.95	0.06	5.1	0.5
Atrazine desethyl	18	35	0.44	0.6	0.83	0.34	1.7	1
Prometryn	—	—	—	—	—	—	—	0.5
Propazine	1	2	0.12	0.12	0.12	0.12	0.12	0.5
Sebuthylazine	—	—	—	—	—	—	—	0.5
Simazine	9	18	0.11	0.17	1.4	0.08	4	0.5
Terbuthylazine	—	—	—	—	—	—	—	0.5
Terbutryn	2	3.9	0.05	0.05	—	0.05	0.06	1
Metamitron	—	—	—	—	—	—	—	0.5
Metribuzin	—	—	—	—	—	—	—	2
Chloridazon	—	—	—	—	—	—	—	0.5
Urea substitutes								
Metoxuron	—	—	—	—	—	—	—	2
1‐(3,4‐Dichlorophenyl)‐3‐methylurea	51	100	0.41	0.59	1.3	0.1	2.7	0.5
1‐(3,4‐Dichlorophenyl)urea	51	100	2.1	3.3	5.5	0.6	25.6	10
3,4‐Dichloroaniline	—	—	—	—	—	—	—	2
Chloroxuron	—	—	—	—	—	—	—	1
Chlortoluron	—	—	—	—	—	—	—	2
Diuron	51	100	0.57	0.98	1.7	0.17	5.6	1
Fenuron	28	55	0.03	0.06	0.12	0.02	2	2
Isoproturon	—	—	—	—	—	—	—	0.5
Linuron	—	—	—	—	—	—	—	1
Methabenzthiazuron	—	—	—	—	—	—	—	0.5
Metobromuron	—	—	—	—	—	—	—	0.5
Monolinuron	—	—	—	—	—	—	—	0.5
Dinitroanilines								
Trifluraline	51	100	0.01	0.02	0.02	0	0.1	0.5
Pendimethalin	—	—	—	—	—	—	—	1
Miscellaneous								
Alachlor	—	—	—	—	—	—	—	0.5
Dimethachlor	—	—	—	—	—	—	—	0.5
Iprodione	—	—	—	—	—	—	—	2
Aclonifen	—	—	—	—	—	—	—	5
Fenhexamid	—	—	—	—	—	—	—	5
Spinosyn A	18	35	0.01	0.02	0.02	0.01	0.06	0.5
Crimidine	—	—	—	—	—	—	—	0.5
Fenarimol	—	—	—	—	—	—	—	0.5
Propargite	—	—	—	—	—	—	—	0.5
Prosulfocarb	51	100	0.06	0.08	0.1	0.04	0.19	0.5
Lenacil	51	100	1.7	1.8	1.9	1.4	2.7	1
DMST	—	—	—	—	—	—	—	0.5
Bisphenol A	51	100	2.2	6.6	13	1.2	91.2	—
Bisphenol S	51	100	3.9	9.4	23.6	0.72	112	10

**FIGURE 4:**
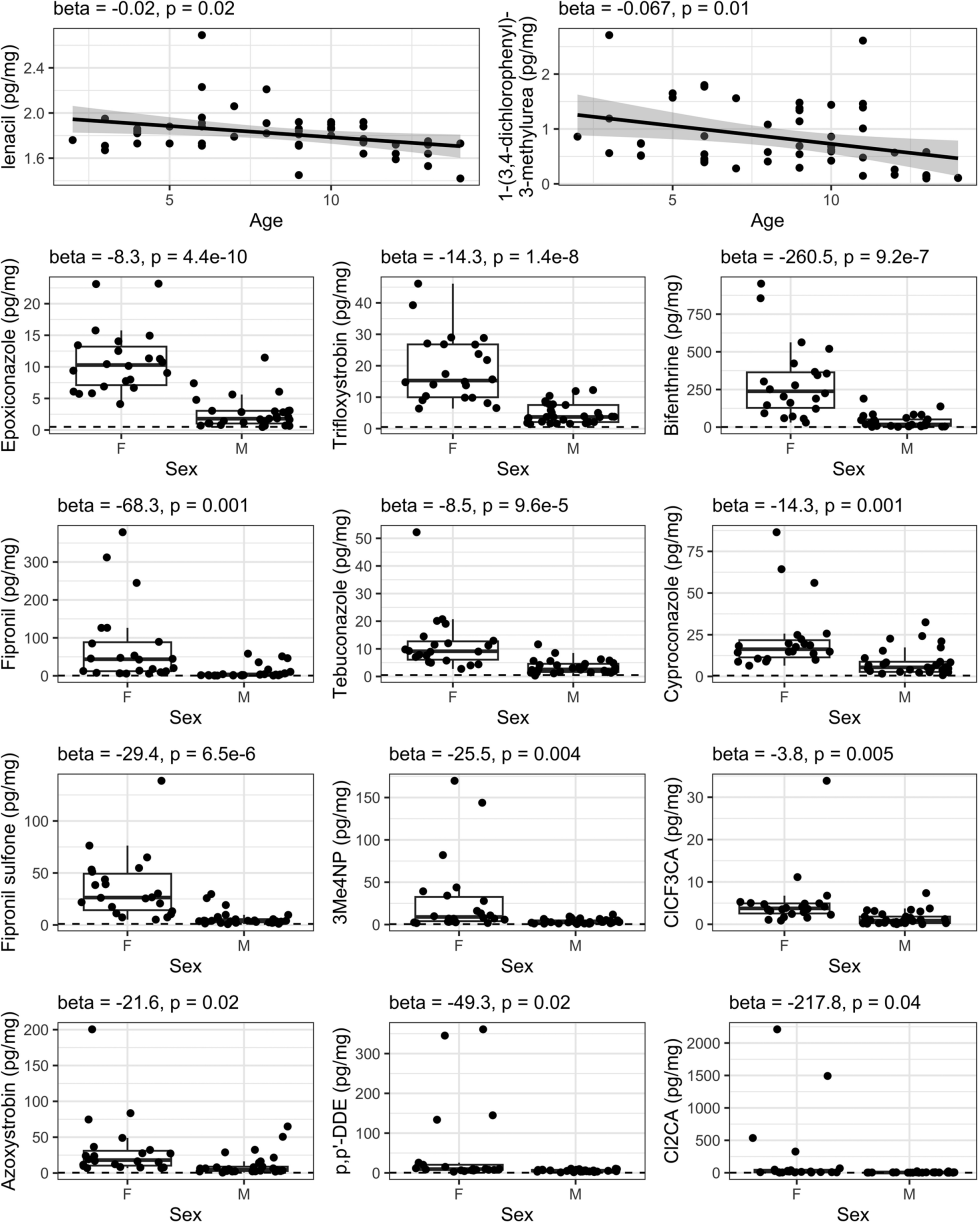
Differences in the environmental exposure to pesticides depending on age and sex (F or M).

To explore demographic influences on exposure, we analyzed the 51 metabolites detected in more than 50% of participants (Figure 4). Only lenacil and 1‐(3,4‐dichlorophenyl)‐3‐methylurea showed a significant negative correlation with age, decreasing by 0.02 pg/mg (*p* = 0.02) and 0.07 pg/mg (*p* = 0.01) per year, respectively. A total of 12 metabolites had their levels higher in females.

## 4 Discussion

The present study identified exposure to multiple environmental pollutants and pesticides from various chemical families in children from a rural community through hair biomonitoring. The study population primarily consisted of farming families whose diet relies heavily on their own agricultural production. These families reside in an area surrounded by extensive soybean crops. Children in this community may be exposed to pesticides through drift or volatilization from applications on soybean fields, domestic use of these chemicals, and through contaminated water and food. It is common for multiple pesticides to coexist in the environment, including in water, soil, air, and food sources [3, 4, 5, 6]. It is likely that synergistic interactions between various pollutants may result in different and more severe effects than those caused by each pollutant individually [22], as demonstrated in laboratory studies [23, 24]. Exposure levels deemed safe for a single pollutant could become harmful when combined with simultaneous exposure to multiple chemicals.

Exposure to multiple chemical substances in hair samples, including pesticides had been reported in several studies carried out in different populations such as Chinese women [22], French mothers, and children [21, 25]. The consistent detection of multiple chemicals across studies underscores growing concern about cumulative chemical exposure as a major environmental health issue.

A previous study in the same community found genotoxic and cytotoxic effects [19]. Prenatal environmental pesticides exposure has been related to lower head circumference at birth [26] and neurodevelopmental alterations at 34 months [27] in a population of the Alto Parana department of Paraguay. Together, these findings highlight the need for targeted epidemiological studies focusing on vulnerable groups such as children, adolescents, and pregnant women.

Approximately one‐quarter of all compounds investigated were detected in every hair sample. This technique allows assessing long‐term exposure more effectively than in blood and urine [13]. The incorporation of hair samples into human biomonitoring methods represents a significant advance in research on exposure to environmental factors from conception and throughout life [28]. However, the relationship between external exposure, systemic absorption, and incorporation into hair is complex and influenced by multiple physiological and environmental factors. Consequently, although hair analysis provides valuable qualitative and semiquantitative information on exposure patterns, it cannot be directly translated into internal dose or health risk estimates.

Among the compounds detected, organophosphates (OPs) were the most prevalent. These compounds are mostly used in insecticides widely used worldwide in agriculture, in homes and for pest control in public health [29]. Exposure to OP can cause acute poisoning [30]. Exposure to OPs was associated with the autism spectrum disorders (ASD) [31], disorders of cognitive function in elementary school [32, 33], and DNA oxidative damage [34]. OPs are not persistent substances and are quickly eliminated in urine [35]. The probability of detecting OP compounds was greater in hair samples than in urine in a population of children of rural community of Spain. The collection, handle, and storage of hair samples is easier than urine [36].

Pyrethroids, found in all samples, are synthetic insecticides commonly used in households and agriculture. Prenatal and early‐life exposure to pyrethroids has been associated with neurodevelopmental delays, behavioral problems, and endocrine disruption [37, 38, 39].

The herbicide 2,4‐dichlorophenoxyacetic acid (2,4‐D) was detected in every sample analyzed. This compound, classified by the International Agency for Research on Cancer (IARC) as possibly carcinogenic to humans [40], is known for its embryotoxic, teratogenic, and neurotoxic properties [41].

Neonicotinoids such as imidacloprid and thiamethoxam, also present in 100% of samples, are widely applied in agriculture. Although effective against crop pests, they also impact nontarget species and biodiversity [42]. Experimental work has demonstrated that mixtures of imidacloprid, fipronil, and glyphosate produce synergistic cytotoxic effects, including oxidative stress, mitochondrial dysfunction, and DNA damage [43].

The study also identified endocrine‐disrupting chemicals (EDCs) such as bisphenol A (BPA) and bisphenol S (BPS). EDCs interfere with hormonal regulation, contributing to reproductive, neurological, and metabolic disorders in adults [44, 45] and children [46, 47] BPA, one of the most widely produced synthetic chemicals, is ubiquitous in plastics, food containers, and consumer goods. Prenatal exposure alters epigenetic programming, potentially predisposing offspring to chronic diseases [48]. Due to its persistence and widespread presence in food, beverages, air, and dust, BPA has been recognized by the European Food Safety Authority (EFSA) as a public health concern for all age groups [49].

The finding of a generally higher pesticide exposure in girls and younger children aligns with previously published observations [50, 51]. The differences in exposure between boys and girls can be hypothesized to stem from distinct gender‐specific activities, hobbies, and food preferences, reflecting the impact of social norms. It is also possible that higher basal metabolic rate in younger children, with air volume intake and food consumption are about four times greater than those of adults for the same weight, can increase exposure levels.

This research is limited by the small sample size and lack of detailed information on nutritional status or neurodevelopmental outcomes. Nonetheless, it represents the first biomonitoring study in Paraguay to characterize children's exposure to multiple pesticides using hair analysis. The results are consistent with findings from other regions [21, 25, 51, 52] and highlight the urgent need for preventive public health measures in agricultural communities.

## 5 Conclusions

This study reveals the simultaneous exposure of children from Colonia San Juan to numerous pesticides and environmental contaminants, including substances with known neurotoxic and endocrine‐disrupting potential. The findings underscore the value of hair biomonitoring as a tool for assessing chronic, real‐world exposure to multiple chemicals. Urgent preventive actions and enforcement of pesticide regulations are needed to protect rural populations, especially children and pregnant women, during critical windows of development and to mitigate the long‐term health risks associated with cumulative chemical exposure.

## Funding

The authors received no specific funding for this work.

## Conflicts of Interest

The authors declare no conflicts of interest.

## Supporting information


**Table S1:** Validation parameters of the methodology used for hair analysis

## Data Availability

The data that support the findings of this study are available from the corresponding author upon reasonable request.
